# Organic food consumption and fecundability in a preconception cohort study of Danish couples trying to conceive

**DOI:** 10.1111/ppe.12924

**Published:** 2022-09-07

**Authors:** Sissel Jessen Weissert, Ellen Margrethe Mikkelsen, Bjarke H. Jacobsen, Elizabeth E. Hatch, Amelia K. Wesselink, Lauren A. Wise, Kenneth J. Rothman, Henrik T. Sørensen, Anne Sofie Dam Laursen

**Affiliations:** ^1^ Department of Clinical Epidemiology Aarhus University and Aarhus University Hospital Aarhus Denmark; ^2^ Department of Epidemiology Boston University School of Public Health Boston Massachusetts USA; ^3^ RTI Health solutions Research Triangle Park North Carolina USA

**Keywords:** diet, fecundability, fertility, organic food, preconception cohort

## Abstract

**Background:**

Little is known about potential health effects of eating organic food in relation to reproduction.

**Objective:**

We examined associations between organic food consumption and fecundability.

**Methods:**

Data were derived from a preconception cohort study of Danish couples trying to conceive (SnartForældre.dk, SF). Participants completed a baseline questionnaire on socio‐demographics, anthropometrics and lifestyle and a validated food‐frequency questionnaire, which included questions on proportions of organic food consumed within six food groups. Participants were followed up with bimonthly questionnaires for up to 12 months or until pregnancy. Analyses were restricted to 2061 participants attempting pregnancy for ≤6 cycles at enrollment and 1303 with <3 cycles. Fecundability ratios (FRs) and 95% confidence intervals (CI) were estimated by proportional probabilities regression models adjusted for potential confounders including age, lifestyle and socioeconomic factors. Associations were examined for vegetables, fruits, cereals, dairy products, eggs and meat, separately, and for the overall pattern of organic food consumption (organic sum score).

**Results:**

The final analytic sample comprised 2069 participants. In the full cohort, organic food consumption was not meaningfully associated with fecundability. Among participants <3 cycles of pregnancy attempt at study entry (*n* = 1303), the FR was 1.11 (95% CI 0.93, 1.33) for the category ‘less than half’, for ‘more than half’ the FR was 1.17 (95% CI 0.99, 1.38) and for ‘almost everything’ the FR was 1.12 (95% CI 0.97, 1.28).

**Conclusion:**

Higher consumption of organic foods was not meaningfully associated with fecundability, although slightly greater fecundability was seen among participants with <3 cycles of pregnancy attempt time.

**Synopsis**

**Study question**

To what extent is organic food consumption associated with fecundability?

**What is already known**

Previous studies have investigated the association between pesticide exposure from fruit and vegetable consumption and fertility, but with inconsistent results.

**What do this study add**

We examined the association between organic food consumption and fecundability for vegetables, fruits, cereals, dairy products, eggs and meat separately and for the overall pattern of organic food consumption.

We constructed an *organic sum score* as a measure of the proportion of organic foods consumed by each woman. We added the absolute intake (g/day) of each of the food groups to the *organic sum score*, to achieve a more accurate measure of organic food consumption.

## 1 INTRODUCTION

Consumers are increasingly purchasing organic foods rather than conventionally farmed foods.^1^ The belief that organic foods are more healthful may contribute to this trend.^2^, ^3^, ^4^ However, evidence is sparse regarding potential health benefits of consuming organic foods.^5^

Several studies suggest that organic foods contain lower levels of environmental contaminants, including pesticide residues, compared with conventionally farmed foods.^5^, ^6^, ^7^, ^8^, ^9^ Some pesticides may, among others, affect fertility by acting as endocrine disruptors, interfering with fertilisation and implantation in women and affecting semen quality in men.^10^, ^11^, ^12^ A diet with lower levels of pesticide residues may, therefore, improve fertility.^13^

Previous studies have investigated organic food consumption and pesticide exposure from food consumption in relation to different reproductive outcomes.^14^, ^15^, ^16^, ^17^, ^18^, ^19^, ^20^ Two studies derived a pesticide residue burden score by combining intake of fruits and vegetables reported on food‐frequency questionnaires with sampling data from the USDA's Pesticide Residue Program.^17^, ^20^ In the first study, a prospective cohort study of 325 women receiving fertility treatment, consumption of vegetables and fruit with high levels of pesticide residues was associated with a lower probability of live birth.^17^ In the second study, a prospective cohort study of 5234 couples trying to conceive spontaneously, there was no appreciable association between consumption of high‐ and low‐pesticide residue fruits and vegetables and fecundability.^20^These studies investigated the association between pesticide exposure from two food groups (i.e. fruit and vegetables) and fertility. In this preconception cohort study of Danish couples trying to conceive, we examined the association between self‐reported intake of organic foods and fecundability for vegetables, fruits, cereals, dairy products, eggs and meat.

## 2 METHODS

### 2.1 Population and study design

The SnartForaeldre.dk Study (Soon Parents, SF) is an ongoing, prospective cohort study of Danish couples trying to conceive. From August 2011 through January 2021, 8559 women enrolled. Participants were invited via social media and a national digital post system (‘e‐books’) with the recruitment area covering all of Denmark.^21^, ^22^ Eligible female participants were between 18 and 49 years old. Additional eligibility criteria were: Danish residence, current relationship with a male partner, trying to conceive and not using fertility treatment.

**FIGURE 1:**
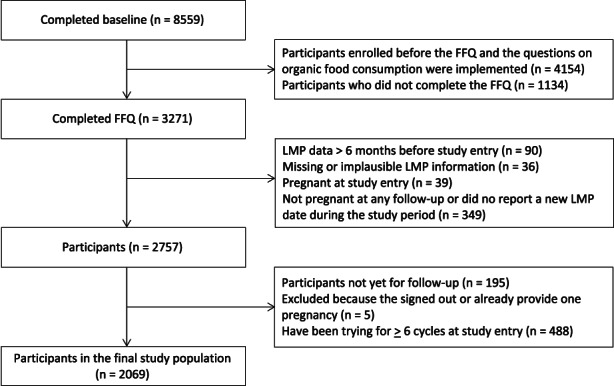
Flowchart of SnartForrældre.dk participants

Dietary intake was estimated using a food‐frequency questionnaire initiated in 2013.^23^ The analytic cohort was restricted to participants enrolled after October 2017, the date we added questions on organic food consumption. Exclusion criteria are shown in Figure 1. The final analytic sample included 2069 female participants.

The cohort complies with Danish regulations for data protection and is registered with Aarhus University (2016‐051‐000001, # 431).

### 2.2 Data sources

Primary data collection was conducted via online self‐administered questionnaires. Eligible participants completed a baseline questionnaire on sociodemographic, lifestyle and behavioural factors as well as reproductive and medical history. Ten days after enrollment, participants completed a food‐frequency questionnaire designed specifically for the study population and validated with respect to food and nutrient intake.^23^ On the food‐frequency questionnaire, participants reported intake of approximately 220 foods and beverages (frequency of standard servings) during the previous year. Participants received bimonthly questionnaires that provided data on pregnancy status, date of last menstrual period and lifestyle factors likely to change over time. Follow‐up continued until pregnancy, start of fertility treatment, loss to follow‐up, withdrawal, cessation of pregnancy attempt or 12 cycles of attempt time, whichever occurred first.

### 2.3 Assessment of organic food consumption

Information on consumption of organic foods was based on six questions on specific food groups included in the food‐frequency questionnaire. These covered total fruits (excluding fruit juice), vegetables, dairy products, bread and cereals, eggs and meat. Participants were asked ‘how much of the food you eat is organic?’. The response options were: ‘I do not eat this type of food’, ‘almost none’, ‘less than half’, ‘more than half’ and ‘almost everything’. Inspired by a previous study,^19^ we constructed an *organic sum score* as a measure of the proportion of organic foods consumed by each woman. In our study, we accounted for the absolute intake (g/day) of each of the food groups in the construction of the organic sum score, to achieve a more accurate measure of organic food consumption. Each response option was given an *organic score* from 0 to 3, where 0 corresponded to ‘almost none’ or ‘I do not eat this type of food’ and 3 corresponded to ‘almost everything’. The organic sum score was calculated using the following formula:

$$\mathrm{OS}S_{j}=\sum_{i=1}^{6}\left(\frac{\text{amoun}t_{i}}{\text{total food amount}}\right)\times OS_{i}$$

in which OSS_*j*_ is the organic sum score for individual *j* and *i* denotes each of the six food groups. Amount_*i*_ is the amount of food group *i* consumed by individual *j* (g/day). Total food amount is the sum of the six food groups (g/day) and OS_*i*_ is individual *j*'s organic score for food group *i*. The contribution from the individual food groups to the total organic sum score was highest for vegetables, cereals, dairy products and fruit (Pearson's correlation coefficients ranged from .65 to .76, Table S1) and lowest for eggs and meat (correlation coefficients: .40 and .46, Table S1). Participants were divided into four groups in which ‘almost none*’* corresponded to an organic sum score < 1, ‘less than half’ corresponded to a score of 1 to <1.5, ‘more than half*’* corresponded to a score of 1.5 to <2 and ‘almost everything’ corresponded to a score ≥2.

To evaluate the extent to which results differed based on classification of the organic sum score, we constructed alternative scores. The results were similar when using these alternative scores (Supplemental text and Tables S2 and S3).

### 2.4 Assessment of TTP

The study endpoint was self‐reported pregnancy. Time to pregnancy was measured in menstrual cycles and calculated using information on cycle length and date of last menstrual period ascertained from baseline and follow‐up questionnaires. Women with regular menstrual cycles were asked to report their usual cycle length. Among women with irregular menstrual cycles, the menstrual cycle length was estimated based on LMP dates reported at baseline and prospectively during follow‐up. Time to pregnancy was estimated in discrete menstrual cycles using the following formula:

$$\text{Time to pregnancy}=\left[\left(\text{cycles of pregnancy attempts}\;\mathrm{at}\;\text{baseline}\right)+\left(\frac{\text{last menstrual period}\;\text{date from most recent follow}\;\mathrm{up}-\text{date of baseline questionnaire}}{\text{usual cycle length}}\right)\right]+1$$

### 2.5 Assessment of covariates

Information on potential confounders was reported at baseline, including: education, monthly household income, last method of contraception, previous pregnancies, intercourse frequency and timing of intercourse, weight, height, smoking status, alcohol consumption, caffeine and sugar‐sweetened beverage intake, use of multivitamins, physical and sedentary activity, woman's age and partner's age. Body Mass Index (BMI) was calculated as kg/m^2^. Physical activity was estimated by calculating total metabolic equivalents per week using the short‐form International Physical Activity Questionnaire, in which all metabolic equivalents hours from walking, moderate physical activity and vigorous physical activity are summed.^24^

### 2.6 Statistical analysis

To account for variation in attempt time at study entry (0–6 cycles) and to avoid left truncation bias, we used the Andersen‐Gill data structure to analyse observed cycles at risk.^25^, ^26^ Fecundability ratios (FRs) and 95% confidence intervals (CI) were estimated using a discrete‐time proportional probabilities regression model.^27^ Indicator terms for ‘cycle at risk’ were included in the model to account for the cohort's declining probability of conception during follow‐up. The FR represents the cycle‐specific probability of conception comparing each of the categories (*less than half, more than half* and *almost everything*) with the reference group (*almost none*). A FR < 1 indicates longer time to pregnancy. In the primary analysis, we used the organic sum score as the exposure variable. In additional analyses, we used the proportion of organic food consumption in each of the six food groups, separately, as the exposure, excluding participants who did not eat the food group under examination.

Potential confounders were selected based on existing literature. The analyses were adjusted for age (<28, 28–32, >32 years [the model did not converge with more groups]), partner's age (<27, 27–29, 30–34, 35–39, ≥40), education (no education, <3, 3–4, >4 years), monthly household income (<25,000, 25,000–39,999, 40,000–65,000, >65,000 DKK), BMI (<25, 25–29, ≥30), metabolic equivalents hours/week (50–59, 60–69, ≥70), current smoking (yes/no), parous (yes/no) and alcohol (none, 1–3, ≥4 servings/week) as categorical variables (Adjusted Model).

### 2.7 Missing data

We multiply‐imputed missing covariate data using fully conditional specification.^28^ We imputed binary variables using logistic regression, ordinal variables using cumulative logistic regression, nominal variables using generalised logistic regression and continuous variables using predictive mean matching. We applied logarithmic transformation for continuous variables that, by visual inspection, appeared non‐normally distributed and where the transformation yielded a better fit. We imputed missing values ordered by missingness, that is variables with the lowest number of missing values were imputed first. We generated 20 imputed data sets, performed the analyses on each individual data set and combined the 20 parameter estimates and confidence intervals into one parameter estimate and confidence interval using Rubin's rule.^29^ Information was missing for <1% of participants for organic food intake, age, smoking status, parity and dietary guidelines. Information was missing for 1–5% of participants for educational attainment, BMI and total metabolic equivalents hours. Information about income was missing for 6% of participants and information about alcohol was missing for 9% of participants. In addition, to reduce selection bias due to differential loss to follow‐up (12%), we assigned one cycle of follow‐up to participants who did not complete any follow‐up questionnaires and multiply‐imputed their outcome (pregnant: yes/no) in that cycle.^30^

### 2.8 Sensitivity analysis

To avoid reverse causation (e.g. if couples with difficulties conceiving began consuming more organic foods to improve their chances of conception), we repeated our analyses among participants with <3 menstrual cycles of attempt time at study entry.

Obesity has been associated with several harmful effects related to fertility, for instance alteration in the uterine environment with enhanced glycated end products, which may impair embryo implantation and thereby compromise pregnancy chances.^31^ Further, fecundability decreases with increasing age.^32^ To assess possible effect measure modification, we performed analyses stratified by age (<30 vs. ≥30 years), BMI (<25 vs. ≥ 25) and parity (parous vs. nulliparous) because organic food consumption may be beneficial only among women whose fertility is not already compromised by these factors. The analyses were adjusted for all variables in the adjusted model. The analysis was conducted on the multiplicative scale.

All statistical analyses were performed using the statistical software SAS 9.4 (SAS Institute Inc.).

### 2.9 Ethics approval

The study was conducted in accordance with the 1964 Declaration of Helsinki and complies with Danish and European regulations about data protection. The study is registered with the Danish Data Protection Agency via Aarhus University (2016‐051‐000001, # 431). Participants provided online informed consent at enrollment.

## 3 RESULTS

### 3.1 Characteristics of the population

The analytic sample comprised 2069 participants who contributed 1491 pregnancies and 7281 menstrual cycles of attempted pregnancy. Among the 2069 participants, 34% reported that ‘almost none’ of the food they consumed was organic, 16% reported ‘less than half’, 17% reported ‘more than half’ and 33% reported that ‘almost all’ food they consumed was organic (Table 1). The median age was slightly higher for participants with an organic sum score ≥ 2 (‘almost everything’) and they had a higher monthly household income and higher educational level compared with those with an organic sum score < 1 (‘almost none’). They were also more likely to be parous and to have regular menstrual cycles. They had a lower BMI and lower intake of sugar‐sweetened beverages. They were also less likely to be current smokers, more likely to take a daily multivitamin and had a higher caffeine intake. Further, women who were more likely to choose the organic alternative within each food group also had a higher intake of vegetables, fruits, eggs, dairy products and cereals, but a lower intake of meats compared with women who did not choose the organic option for the respective food group (Table 2).

**TABLE 1:** Baseline characteristics of 2069 participants

	Proportion of organic food consumption in the overall food intake (organic sum score)				All
	Almost none	Less than half	More than half	Almost everything	
Number of women, *n* (%)	699 (33.8)	323 (15.6)	356 (17.2)	691 (33.4)	2069 (100)
Organic sum score (mean)	0.35	1.24	1.76	2.57	1.47
Age, years, median (P10 P90)	29.2 (26.4 33.6)	30 (26.7 34.4)	29.7 (27.2 34.0)	30.6 (26.9 34.7)	29.9 (26.7 34.2)
Partner's age, years, median (P10 P90)	31 (26 38)	32 (27 38)	32 (27 39)	32 (27 38)	32 (27 38)
Total household income/month DKK (%)					
<39,999	27.3	18.3	24.2	19.5	22.8
40,000–65,000	44.6	41.2	34.6	37	39.8
65,000+	28	40.6	41.3	43.4	37.4
Higher education (%)					
4 or less years	64.1	54.5	48.3	43.6	53
>4 years	35.9	45.5	51.7	56.4	47
BMI, kg/m^2^, median (P10, P90)	24.1 (20.2, 34.2)	23.2 (19.5, 31.2)	23.6 (19.8, 30.8)	22.2 (19.3, 27.7)	23.2 (19.7, 31.1)
Cycles of attempt at study entry, *n* (%)					
<3 cycles	424 (60.7)	188 (58.2)	229 (64.3)	462 (66.9)	1303 (62.9)
3–6 cycles	275 (39.3)	135 (41.8)	127 (35.7)	229 (33.1)	766 (37.1)
MET hours/week, median (P10, P90)	39.8 (9.3, 158.6)	39.4 (10.5, 151)	38.1 (10.3, 130.2)	39.9 (11.6, 145.3)	39.4 (10.5, 148.5)
Low adherence to Danish Dietary guidelines (%)	36.8	26.6	27.5	17.7	27.2
Current smoker, yes (%)	11.4	10.2	9.3	11.3	10.8
Female alcohol beverage, drinks/week, median (P10 P90)	1.0 (0.0 5.5)	2.0 (0.0 6.0)	2.0 (0.0 6.0)	2.0 (0.0 6.0)	2.0 (0.0 6.0)
Caffeine intake, mg/day, median (P10, P90)	88.4 (0.0, 434.5)	157.2 (7.6, 449.1)	163.8 (19.7, 471.6)	185.4 (11.8, 475.0)	157.2 (7.6, 471.3)
Sugar‐sweetened beverages including juice, drinks/week, median (P10, P90)	1.5 (0.5, 5.0)	1.5 (0.5, 4.5)	1.5 (0.5, 4.5)	1.0 (0.5, 3.5)	1.5 (0.5, 4.0)
Daily multivitamin intake, yes (%)	51.8	53.9	52.2	57.9	54.2
Parous (%)	33.2	27.9	33.7	43.1	35.8
Regular cycles, yes (%)	73.2	74	70.8	77.1	74.2
Last method of contraception (%)					
Hormonal	58.7	59.8	54.2	45.9	53.8
Barrier methods/rhythm/withdrawal/other	41.3	40.2	45.8	54.1	46.2

**TABLE 2:** Food consumption (g/day) of each of the six food groups; total intake among all participants and intake presented by response categories of organic food consumption

Food category	Number observations, *n*	Amount consumed (g/day)		
		10th Pctl.	Median	90th Pctl.
Vegetables				
All participants^a^	2069	126.6	275.8	555.3
Almost none^b^	292	101.0	220.5	509.9
Less than half^b^	604	119.6	249.0	502.0
More than half^b^	665	142.0	291.8	555.3
Almost everything^b^	459	166.0	337.4	654.5
Fruits				
All participants	2069	36.6	107.2	282.7
Almost none	400	29.4	96.1	264.9
Less than half	654	35.9	100.8	280.7
More than half	517	41.2	111.1	289.3
Almost everything	424	46.6	125.0	295.4
Meat				
All participants	2069	27.5	68.2	123.1
Almost none	707	42.3	76.5	129.1
Less than half	670	37.2	70.7	125
More than half	295	32.3	65.5	115
Almost everything	201	18.1	49.5	97.3
Eggs				
All participants	2069	10.4	22	59
Almost none	372	10.1	20.2	59.1
Less than half	277	10.5	22.1	58
More than half	248	10.4	21.7	58.8
Almost everything	1069	11	22.8	62.3
Bread and cereals				
All participants	2069	97.2	174.5	284.0
Almost none	632	90.7	169.3	269.1
Less than half	658	100.1	175.0	289.9
More than half	401	100.4	182.2	285.2
Almost everything	261	101.7	181.8	285.5
Dairy products				
All participants	2069	120.8	315.0	712.4
Almost none	473	123.8	305.4	724.6
Less than half	348	143.6	333.3	720.8
More than half	379	126.8	321.8	706.7
Almost everything	739	131.5	330.1	726.8

^a^ Intake of any vegetables, fruits, meats eggs, bread and cereals, dairy products in the full analytic sample.

^b^ Participants who chose the response option; ‘’almost none”, ”less than half”, ”more than half” or ”almost everything” to the question in the FFQ: how much of the food you eat is organic?

We excluded 1134 participants who did not complete the food‐frequency questionnaire. Compared to those included in the analytic cohort, excluded women had less education, lower household income, slightly higher BMI and were more likely to be current smokers. They also had lower intake of caffeine, alcohol and sugar‐sweetened beverages (Table S4).

### 3.2 Consumption of organic food and fecundability

In the full cohort, higher organic sum score was associated with a slightly higher probability of pregnancy in the unadjusted model (Table 3). However, we did not observe a monotonic pattern, and the estimate attenuated after adjustment for covariates (Table 3, Adjusted Model). Among participants <3 cycles of pregnancy attempt at study entry (*n* = 1303), higher consumption of organic foods was associated with a slightly higher probability of pregnancy; for the category ‘less than half’ the FR was 1.11 (95% CI: 0.93, 1.33), for ‘more than half’ the FR was 1.17 (95% CI: 0.99, 1.38) and for ‘almost everything’ the FR was 1.12 (95% CI: 0.97, 1.28), (Table 3, Adjusted Model).

**TABLE 3:** Organic food consumption and fecundability

Organic food group	Full cohort (attempt time at study entry 0–6 cycles)				Attempt time at study entry <3 cycles		
	Pregnancies, *n*	Cycles, *n*	Unadjusted model	Adjusted model^a^	Pregnancies, *n*	Cycles, *n*	Adjusted model^a^
			FR (95% CI)	FR (95% CI)			FR (95% CI)
Organic sum score							
Almost none (ref)	488	2560	1.00 (reference)	1.00 (reference)	313	1658	1.00 (reference)
Less than half	226	1188	1.00 (0.87, 1.16)	1.00 (0.86, 1.15)	145	714	1.11 (0.93, 1.33)
More than half	268	1184	1.13 (0.99, 1.29)	1.10 (0.96, 1.26)	183	768	1.17 (0.99, 1.38)
Almost everything	509	2349	1.10 (0.98, 1.22)	1.02 (0.91, 1.14)	370	1570	1.12 (0.97, 1.28)
Vegetables							
Almost none	209	1058	1.00 (reference)	1.00 (reference)	128	643	1.00 (reference)
Less than half	435	2128	1.02 (0.89, 1.18)	1.02 (0.88, 1.18)	278	1380	1.04 (0.86, 1.25)
More than half	474	2359	0.99 (0.86, 1.14)	0.97 (0.83, 1.12)	341	1516	1.09 (0.90, 1.31)
Almost everything	337	1566	1.05 (0.90, 1.22)	0.97 (0.83, 1.13)	244	1085	1.03 (0.85, 1.25)
Fruit							
Almost none	288	1411	1.00 (reference)	1.00 (reference)	178	881	1.00 (reference)
Less than half	469	2340	0.98 (0.86, 1.11)	0.95 (0.84, 1.09)	308	1497	1.00 (0.84, 1.18)
More than half	360	1877	0.93 (0.81, 1.07)	0.88 (0.76, 1.01)	263	1236	0.98 (0.82, 1.16)
Almost everything	316	1411	1.06 (0.93, 1.22)	0.95 (0.83, 1.10)	228	962	1.02 (0.86, 1.22)
Meat							
Almost none	498	2592	1.00 (reference)	1.00 (reference)	320	1727	1.00 (reference)
Less than half	480	2321	1.07 (0.96, 1.19)	1.03 (0.92, 1.16)	332	1459	1.17 (1.02, 1.34)
More than half	225	990	1.13 (0.99, 1.30)	1.07 (0.93, 1.23)	164	665	1.17 (0.99, 1.39)
Almost everything	147	701	1.04 (0.88, 1.22)	1.01 (0.85, 1.19)	105	476	1.08 (0.89, 1.32)
Eggs							
Almost none	253	1368	1.00 (reference)	1.00 (reference)	160	872	1.00 (reference)
Less than half	201	979	1.11 (0.94, 1.31)	1.10 (0.93, 1.30)	136	618	1.20 (0.98, 1.48)
More than half	173	875	1.10 (0.93, 1.31)	1.05 (0.89, 1.26)	111	510	1.13 (0.91, 1.40)
Almost everything	791	3703	1.11 (0.99, 1.27)	1.07 (0.94, 1.22)	557	2499	1.13 (0.96, 1.33)
Bread and cereals							
Almost none	439	2348	1.00 (reference)	1.00 (reference)	289	1559	1.00 (reference)
Less than half	475	2257	1.11 (0.99, 1.25)	1.11 (0.99, 1.25)	321	1429	1.19 (1.03, 1.38)
More than half	293	1318	1.14 (1.00, 1.30)	1.11 (0.97, 1.27)	206	842	1.19 (1.01, 1.39)
Almost everything	199	961	1.07 (0.92, 1.24)	1.02 (0.88, 1.19)	146	687	1.09 (0.91, 1.30)
Dairy products							
Almost none	324	1716	1.00 (reference)	1.00 (reference)	207	1107	1.00 (reference)
Less than half	243	1276	1.01 (0.87, 1.17)	1.00 (0.86, 1.16)	154	792	1.05 (0.87, 1.27)
More than half	280	1321	1.08 (0.94, 1.24)	1.06 (0.91, 1.22)	199	900	1.13 (0.95, 1.36)
Almost everything	553	2489	1.13 (1.00, 1.28)	1.07 (0.94, 1.21)	391	1648	1.12 (0.96, 1.31)

*Note*: Values in parentheses are 95% CIs.

Abbreviation: FR, fecundability ratio.

^a^ Adjusted Model: Adjusted for age, partner's age, vocational training, BMI, total household income, MET hours, smoking status, alcohol intake, parous.

We did not observe any meaningful association between organic food consumption and fecundability when we analysed the six food groups separately (Table 3, Adjusted Model). However, when we restricted the analyses to women with <3 cycles of pregnancy attempt at study entry, the estimates showed a modest association between organic food consumption and fecundability in all food groups except for fruits, especially when comparing ‘more than half’ with ‘almost none’(Table 3, Attempt time at study entry <3 cycles, Adjusted Model).

In the full cohort, higher organic sum score was associated with greater fecundability among participants aged <30 years, BMI < 25 and parous participants, but not among participants aged ≥30 years, BMI ≥ 25 and nulliparous (Table 4). However, the associations were imprecise (Table 4).

**TABLE 4:** Organic food consumption and fecundability stratified by BMI, age and parity

Organic sum score	Full cohort (attempt time at study entry 0–6 cycles)			Attempt at study entry <3 cycles, *n* = 1303		
	Pregnancies, *n*	Cycles, *n*	Adjusted model^a^	Pregnancies, *n*	Cycles, *n*	Adjusted model^a^
			FR (95% CI)			FR (95% CI)
BMI^a^						
<25, *n* = 1383				*n* = 909		
Almost none (ref)	295	1532	1.00 (reference)	197	1031	1.00 (reference)
Less than half	151	800	0.99 (0.83, 1.19)	109	538	1.10 (0.89, 1.36)
More than half	178	708	1.16 (0.98, 1.37)	123	504	1.16 (0.94, 1.42)
Almost everything	395	1752	1.09 (0.95, 1.25)	288	1191	1.16 (0.98, 1.36)
≥25, *n* = 686				*n* = 394		
Almost none (ref)	193	1028	1.00 (reference)	116	627	1.00 (reference)
Less than half	75	388	1.12 (0.87, 1.44)	36	176	1.31 (0.92, 1.86)
More than half	90	476	1.03 (0.81, 1.31)	60	264	1.26 (0.94, 1.70)
Almost everything	114	597	0.91 (0.73, 1.13)	82	379	1.00 (0.76, 1.33)
Age^b^						
<30 years, *n* = 1054				*n* = 678		
Almost none (ref)	279	1596	1.00 (reference)	190	1087	1.00 (reference)
Less than half	108	596	1.02 (0.83, 1.26)	72	378	1.13 (0.88, 1.45)
More than half	139	659	1.12 (0.93, 1.36))	98	445	1.19 (0.95, 1.50)
Almost everything	211	1046	1.11 (0.94, 1.31)	156	701	1.26 (1.03, 1.53)
≥30 years, *n* = 1015				*n* = 625		
Almost none (ref)	209	964	1.00 (reference)	123	571	1.00 (reference)
Less than half	118	593	0.95 (0.78, 1.16)	73	336	1.05 (0.82, 1.36)
More than half	129	525	1.08 (0.88, 1.32)	85	323	1.12 (0.88, 1.44)
Almost everything	298	1303	0.96 (0.81, 1.12)	214	869	0.99 (0.81, 1.21)
Parity^c^						
Parous, *n* = 740				*n* = 521		
Almost none (ref)	168	784	1.00 (reference)	114	538	1.00 (reference)
Less than half	69	278	1.13 (0.89, 1.45)	50	186	1.27 (0.95, 1.71)
More than half	100	353	1.17 (0.94, 1.46)	75	251	1.22 (0.94, 1.58)
Almost everything	245	812	1.16 (0.97, 1.39)	189	627	1.15 (0.93, 1.43)
Nulliparous, *n* = 1329				*n* = 782		
Almost none (ref)	318	1789	1.00 (reference)	198	1113	1.00 (reference)
Less than half	159	897	0.97 (0.81, 1.16)	96	535	1.04 (0.83, 1.31)
More than half	168	831	1.07 (0.90, 1.28)	108	517	1.14 (0.92, 1.43)
Almost everything	264	1537	0.94 (0.80, 1.10)	181	943	1.08 (0.89, 1.30)

^a^ BMI: Adjusted for age, partner's age, vocational training, BMI (strata BMI ≥ 25 adjusted for the categories 25–29 and ≥30), total household.

^b^ Age: Adjusted for age (two categories in each strata; [<28 and 28–29 years] and [30–32 and >32 years]), partner's age, vocational training, BMI, total household income, MET hours, smoking status, alcohol intake, parous.

^c^ Parity; Adjusted for age, partner's age, vocational training, BMI, total household income, MET hours, smoking status, alcohol intake.

Among participants with <3 cycles of pregnancy attempt at study entry, we observed differences in associations when we stratified by age and parity, but not BMI. Higher organic score was associated with slightly greater fecundability primarily among participants aged <30 years and among parous participants. However, we did not observe a monotonic pattern and the associations were imprecise.

## 4 COMMENTS

### 4.1 Principal findings

In this preconception cohort study, total organic food consumption was not associated with fecundability overall. However, among women with <3 cycles of attempt time at study entry, greater organic food consumption was associated with slightly higher fecundability. To the extent that increasing pregnancy attempt time is associated with behaviour change (e.g. greater intake of organic foods), we would expect results among those with <3 cycles of attempt time to be less prone to reverse causation.

The estimates differed by age and parity. Hence, a beneficial effect of organic food intake was observed among women <30 years and for parous women. Organic food consumption may be beneficial only among women whose fertility is not already compromised due to advanced age^33^, ^34^ and among those with proven fecundity (parous) and thereby no underlying infertility.

### 4.2 Strengths of the study

We constructed the organic sum score as a function of the amount of organic food (g/day) consumed in each of the six food groups. Ascertaining data on the amount of food within each food group is crucial for the validity of the organic sum score because the amount of food consumed in each of the six food groups may not be the same for all participants. Hence, choosing almost everything as organic produce within a given food group may not represent the same exposure to potential contaminants for all participants.

The prospective study design, with enrollment during preconception, reduces the potential for (1) selection bias due to preclusion of couples who never achieve a pregnancy and (2) information bias due to inaccurate recall of time to pregnancy and exposure and covariate information. Further, we were able to adjust our analyses for sociodemographic and behavioural predictors of misclassification of diet that may also be related to fecundability, such as BMI, age and education.^37^, ^38^ However, since psychosocial predictors, such as social desirability, were not measured, non‐differential misclassification is expected. Non‐differential misclassification could cause bias towards or away from the null.^39^, ^40^

We collected data on and adjusted for a wide range of potential confounders, although, residual confounding from participant and partner characteristics was possible. For example, we had limited data on partner diet, and given that male factors account for as much as 50% of subfertility,^41^ the degree of residual confounding could have been large if male diet was an important determinant of fecundability and was strongly associated with partner's organic sum score values.

Participant characteristics differed between women in the analytic sample and women who did not respond to the food‐frequency questionnaire. We expect that factors related to questionnaire completion are mainly sociodemographic and behavioural, such as education, smoking, BMI and alcohol intake. Because such factors may also be confounders of the studied associations, we have adjusted for them in our analyses and at the same time indirectly accounted for their influence on the selection of participants into the analytic sample.

### 4.3 Limitations of the data

An important study limitation is our indirect measurement of organic food consumption. If we had had access to biological material, for example blood and urine levels of pesticides after metabolism and were able to objectively assess pesticide residues, we would have been able to make stronger inferences. Instead, the exposure variable was based on questionnaire data in which the response options ‘almost none’, ‘less than half’, ‘more than half’ and ‘almost everything’ may have been interpreted differently among participants. This could have introduced exposure misclassification. Furthermore, while the food‐frequency questionnaire was validated within the cohort, the question about organic food consumption was added later and not part of the validation.

Each food item may contain specific pesticide molecules and families.^35^ Hence, the benefit of eating organic food may vary based on what individual foods constitute the diet. For example, eating organic foods may have little effect on the body burden of pesticides among participants who mainly consume foods with low potential for pesticide contamination, whereas organic diets may have stronger effects among participants who eat foods with high potential for pesticide contamination, such as strawberries and cucumber.^36^ The organic sum score would have been a better measure of pesticide exposure if we had been able to account for differences in the types and amounts of contaminants across the food groups. This was not possible with the data at hand.

### 4.4 Interpretation

Directly comparing previous studies with each other and with our results is difficult, because of differences in methods of exposure assessment, outcome and study cohort. For instance, the U.S. Environment and Reproductive Health (EARTH) cohort study^17^ and the North American preconception study, Pregnancy Study Online (PRESTO),^18^ have used a Pesticide Residue Burden Score to assess intake of pesticide residues from fruits and vegetables. The EARTH study found an association between high consumption of high‐pesticide residue fruits and vegetables and lower odds of clinical pregnancy among women receiving fertility treatment,^15^ although, their results may be influenced by underlying infertility conditions. For example, women with known subfertility may have changed their diet to improve their fertility. PRESTO studied couples trying to conceive spontaneously and found little association between intake of high‐pesticide residue fruits and vegetables and fecundability. They also examined the association between reported consumption of organic fruits and vegetables and fecundability. Consistent with our results on organic fruit and vegetable intake, there was little association between consumption ‘most of the time’ and fecundability.

Organic foods are produced without the use of synthetic pesticides. This may be the main health benefit distinguishing them from conventionally farmed foods.^42^ Pesticides are a heterogeneous group of chemicals that could have a range of effects in the human body.^35^ Pesticide use varies by crop, region and time period.^43^ All six food groups examined in our study may be potential sources of pesticide residue. However, vegetables, fruits and cereals are thought to account for more than 60% of all pesticide residues in the human body, with conventionally grown fruits and vegetables representing the main sources.^8^, ^36^ Although, we may anticipate a beneficial effect of consuming organic varieties instead of conventional varieties of these food groups, we did not find any appreciable association between the organic sum score and fecundability when investigating fruit and vegetables as individual exposures.

The use of pesticides in food production in Europe, and especially in Denmark, is highly regulated, keeping the allowed amounts of pesticides in agriculture below levels that are expected to be harmful for health, which may explain our results.^1^ Further, organic foods and conventionally grown foods differ in other ways than the use of pesticides, which may affect fertility. Organic foods are also produced without application of synthetic fertilisers, genetically modified organisms or use of antibiotics in animals.^9^ Further, previous studies have found that organic foods contain lower levels of toxic metabolites, including heavy metals such as cadmium and increased levels of omega‐3‐fatty acids in eggs and dairy products, improved fatty acid profiles in organic meat products and higher antioxidant concentrations in organic crops.^13^ However, investigations on any link between these food sources and fertility remain sparse.

## 5 CONCLUSIONS

Overall, we found no meaningful association between fecundability and consumption of organic foods, although there was slightly higher fecundability among participants who had been attempting pregnancy for fewer than three cycles at study entry.

## AUTHOR CONTRIBUTIONS

ASDL and EMM designed the study. EMM, HTS, EEH, LAW and KJR planned and initiated SnartForældre.dk. SJW and BHJ analyzed the data. All authors interpreted the results. SJW drafted the manuscript. All authors approved the final version of the manuscript.

## CONFLICT OF INTEREST

Dr. Wise is a consultant on uterine fibroids and abnormal uterine bleeding for AbbVie, Inc. In the past three years, Dr. Wise has accepted in‐kind donations for primary data collection for Pregnancy Study Online from FertilityFriend.com (fertility apps), Kindara.com (fertility apps), Sandstone Diagnostics (semen tests), LabCorp (semen tests) and Swiss Precision Diagnostics (home pregnancy tests).

## Funding information

Snartforældre.dk was supported by National Institute of Child Health and Human Development (grant number: R01‐HD086742).

## Supporting information


Appendix S1

## Data Availability

To comply with Danish and European regulations, data used in this article will not be made publicly available. Data may be made available to researchers pending on application to and approval by the SnartForældre.dk principal investigators (info@snartforældre.dk).
