# Supplementary material for: Organic food consumption and fecundability in a preconception cohort study of Danish couples trying to conceive
Source: Paediatr Perinat Epidemiol. 2022 Sep 7;37(1):57–68. doi: 10.1111/ppe.12924 (PMC10087289; doi:10.1111/ppe.12924)
Supplement: Supplementary file 1 — Appendix S1 [file PPE-37-57-s001.docx]

**Supplemental material**

**Supplemental text 1**

We constructed two alternative scores to evaluate the robustness of the organic sum score. In the first alternative score, instead of assuming equal distance between the categories, we assumed “almost none” to be 5%, “less than half” to be 40%, “more than half” to be 60% and “almost everything” to be 80%. We chose 80% for the highest category, because even if a person actively wishes to buy everything as organic produce, it may not be possible as not all food products are available as organic produce or in all stores. This Interpretation of the categories yielded a higher alternative organic sum score than the original organic sum score (eTable 2). We also constructed an organic sum score, based on the original scoring with equal distance between categories, where we weighted the food groups differently. Fruit, vegetables and cereals were given 100% contribution, while eggs, meat and dairy products were given 50% contribution. This construction yielded and lower organic sum score for some and a higher organic sum score for others, compared with the original scoring (eTable 2). The associations between the alternative scores and fecundability were not much different from those seen with the original score (eTable 3).

**Supplemental tables**

| **Supplemental Table 1 (eTable 1)**  Pearson’s correlation coefficients of the Organic Sum Score and its individual components | | | | | | | |
| --- | --- | --- | --- | --- | --- | --- | --- |
|  | **Organic sum score** | **Dairy products** | **Meat** | **Eggs** | **Bread and cereals** | **Vegetables** | **Fruits** |
| **Organic sum score** | 1.00 | 0.73 | 0.47 | 0.40 | 0.73 | 0.76 | 0.65 |
| **Dairy products** | 0.73 | 1.00 | 0.27 | 0.19 | 0.38 | 0.20 | 0.24 |
| **Meat** | 0.47 | 0.27 | 1.00 | 0.27 | 0.38 | 0.29 | 0.23 |
| **Eggs** | 0.40 | 0.19 | 0.27 | 1.00 | 0.27 | 0.31 | 0.20 |
| **Bread and cereals** | 0.73 | 0.38 | 0.38 | 0.27 | 1.00 | 0.53 | 0.45 |
| **Vegetables** | 0.76 | 0.20 | 0.29 | 0.31 | 0.53 | 1.00 | 0.54 |
| **Fruits** | 0.65 | 0.24 | 0.23 | 0.20 | 0.45 | 0.54 | 1.00 |

| **Supplemental Table 2 (eTable 2)**  Cross-tabulation of participants by categories of original score vs alternative organic sum score | | | | | |
| --- | --- | --- | --- | --- | --- |
|  | **Alternative score, different distance between categories** | | | | |
| **Original score** | **Almost none** | **Less than half** | **More than half** | **Almost everything** | |
| **Almost none** | 581 | 118 | 0 | 0 |  |
| **Less than half** | 0 | 149 | 174 | 0 |  |
| **More than half** | 0 | 0 | 192 | 164 |  |
| **Almost everything** | 0 | 0 | 0 | 691 |  |
| **Total** | 581 | 267 | 366 | 855 |  |
|  | **Alternative score, down-weighted meat, eggs and dairy** | | | | |
| **Almost none** | 662 | 37 | 0 | 0 |  |
| **Less than half** | 82 | 233 | 8 | 0 |  |
| **More than half** | 4 | 164 | 186 | 2 |  |
| **Almost everything** | 0 | 29 | 267 | 395 |  |
| **Total** | 748 | 463 | 461 | 397 |  |

| **Supplemental Table 3 (eTable 3)**  Fecundability ratios for the alternative Organic Sum Scores | | | | |
| --- | --- | --- | --- | --- |
|  |  |  | **Unadjusted** | **Adjusted** |
|  | **Pregnancies** | **Cycles at risk** | **FR (95% CI)** | **FR (95% CI)** |
| **Different distance between categories** |  |  |  |  |
| Almost none | 404 | 2125 | 1.00 (reference) | 1.00 (reference) |
| Less than half | 190 | 975 | 1.03 (0.88, 1.20) | 1.02 (0.88, 1.20) |
| More than half | 270 | 1233 | 1.10 (0.96, 1.26) | 1.08 (0.94, 1.24) |
| Almost everything | 627 | 2948 | 1.08 (0.97, 1.21) | 1.02 (0.91, 1.14) |
| **Down-weighted meat, eggs and dairy** |  |  |  |  |
| Almost none | 523 | 2751 | 1.00 (reference) | 1.00 (reference) |
| Less than half | 338 | 1595 | 1.09 (0.97, 1.23) | 1.07 (0.94, 1.21) |
| More than half | 333 | 1561 | 1.08 (0.96, 1.22) | 1.03 (0.91, 1.16) |
| Almost everything | 297 | 1374 | 1.10 (0.97, 1.25) | 1.04 (0.92, 1.19) |

*^a^*Adjusted Model: Adjusted for age, partners age, vocational training, BMI, total household income, MET hours, smoking status, alcohol intake, parous

| **Supplemental Table 4 (eTable 4)**  Characteristics of SnartForældre.dk participants who did not respond to the food frequency questionnaire invitation. | |
| --- | --- |
|  | **All** |
| **Number of women, n (%)** | 1134 (100) |
| **Age, years, median (P10 P90)** | 30.2 (26.8 35.0) |
| **Partner’s age, years, median (P10 P90)** | 34.0 (30.0 41.0) |
| **Total household income/month DKK (%)** |  |
| < 39,999 | 27.4 |
| 40,000-65,000 | 36.8 |
| 65,000+ | 26.8 |
| **Higher education (%)** |  |
| 4 or fewer years | 67.8 |
| > 4 years | 28.8 |
| **BMI, kg/m^2^, median (P10 P90)** | 24.6 (19.8 37.5) |
| **MET-hours/week, median (P10 P90)** | 43.8 (8.3 176.4) |
| **Current smoker, yes (%)** | 21.8 |
| **Female alcohol beverage, drinks/week, median (P10 P90)** | 1 (0 5.5) |
| **Caffeine intake, mg/day, median (P10 P90)** | 118.3 (7.6 464.3) |
| **Sugar sweetened beverages including juice, drinks/week, median (P10 P90)** | 1.0 (0 5) |
| **Daily multivitamin intake, yes (%)** | 43.1 |
| **Parous (%)** | 40.4 |
| **Last method of contraception (%)** |  |
| Hormonal | 55.4 |
| Barrier methods/rhythm  /withdrawal/other | 40.2 |
